# Evaluation of copper chaperone ATOX1 as prognostic biomarker in breast cancer

**DOI:** 10.1007/s12282-019-01044-4

**Published:** 2020-01-02

**Authors:** Stéphanie Blockhuys, Donita C. Brady, Pernilla Wittung-Stafshede

**Affiliations:** 1 Department of Biology and Biological Engineering, Chalmers University of Technology, 412 96 Gothenburg, Sweden; 2 Department of Cancer Biology, Perelman School of Medicine, University of Pennsylvania, Philadelphia, PA 19104 USA; 3 Perelman School of Medicine, Abramson Family Cancer Research Institute, University of Pennsylvania, Philadelphia, PA 19104 USA

**Keywords:** ATOX1, Breast cancer, Survival, METABRIC, Prognostic, Biomarker

## Abstract

Copper is involved in different hallmarks of cancer, including metastasis, but responsible copper-binding proteins and pathways are not clear. The copper chaperone ATOX1 was recently shown to play a role in breast cancer cell migration, which is a key step in metastasis. Since most cancer-related deaths are due to metastasis, we hypothesized that *ATOX1* mRNA expression may be associated with breast cancer disease progression and thus, a prognostic biomarker in breast cancer. We therefore studied the association of *ATOX1* expression levels with clinicopathological parameters and survival for 1904 breast cancer patients using the METABRIC data set. Our results indicate *ATOX1* expression levels as a potential prognostic biomarker for ER-positive subtypes and early stages of breast cancer. Pre-clinical studies and clinical trials are desired to identify the molecular roles of ATOX1 in these conditions.

**Electronic supplementary material:**

The online version of this article (10.1007/s12282-019-01044-4) contains supplementary material, which is available to authorized users.

## Introduction

Since copper (Cu) is a key component of many enzymes [1, 2], it is not surprising that Cu is required for at least three characteristic phenomena involved in cancer: proliferative immortality, angiogenesis and metastasis. In support of increased Cu demand, cancer tissue and cancer patients’ serum have increased Cu levels [3] and breast cancers patients with distant metastasis were recently shown to have increased serum Cu levels [4].

Previously, we identified overexpressed Cu transport proteins in cancer, through analysis of the RNA transcript level changes of the whole Cu proteome (i.e., for 54 identified Cu-binding proteins) in different cancer tissues using information from The Cancer Genome Atlas, or TCGA, database. Our analysis revealed that, with respect to ATOX1, it is upregulated in breast, colorectal, uterus and liver tumors, and the breast cancer data were confirmed by us in tissue microarray experiments [5]. Moreover, when imaging cells, we found ATOX1 to be localized at lamellipodia edges of aggressive breast cancer cells. This hinted to a role in cancer cell migration and, indeed, upon *ATOX1* silencing, wound closure was reduced [6]. Since cancer metastasis consists of a cascade of processes that depends sensitively on cell migration this implies that ATOX1 may be important for processes facilitating metastasis [7]. Importantly, 90% of all cancer-related deaths are due to metastasis [8].

To assess the putative role of ATOX1 as a prognostic biomarker in breast cancer patients, we here investigated the correlation between *ATOX1* mRNA expression levels and patient survival using the Molecular Taxonomy of Breast Cancer International Consortium (METABRIC) breast cancer database. METABRIC covers a large variety of breast cancers with long-term follow-up data, while other large-scale data sets like TCGA are limited in the analysis of clinical associations by the scarcity of long-term patient follow-up data and stringent criteria used for sample selection.

## Materials and methods

### METABRIC

Clinical data and *ATOX1* mRNA expression z-scores were extracted from the METABRIC breast cancer study database publicly available via cBioPortal website, https://www.cbioportal.org/ [9–11]. The mRNA expression scores were available for 1904 primary breast cancers, out of the total number of 2509 patients, with a maximum follow-up period of 355 months. Detailed information about tissue collection and staging is described by Curtis et al*.* [9].

### Statistics

For data analysis cutoff was arbitrarily set at 10%, meaning “Low” are the bottom 10% and “high” are the top 10% of the patient cohort after sorting patients based upon *ATOX1* mRNA expression levels. Statistical analysis was performed using SPSS software (IBM SPSS statistics version 22). Patient survival was plotted using Kaplan–Meier (KM) curves with log-rank statistical test. The Chi-square test was used to analyze the correlation between clinicopathological parameters and *ATOX1* mRNA expression scores. COX proportional hazard model was applied to analyze the correlation between clinicopathological parameters and disease-specific survival (DSS).

## Results

### High *ATOX1* expression is associated with poor survival of breast cancer patients

**Fig. 1:**
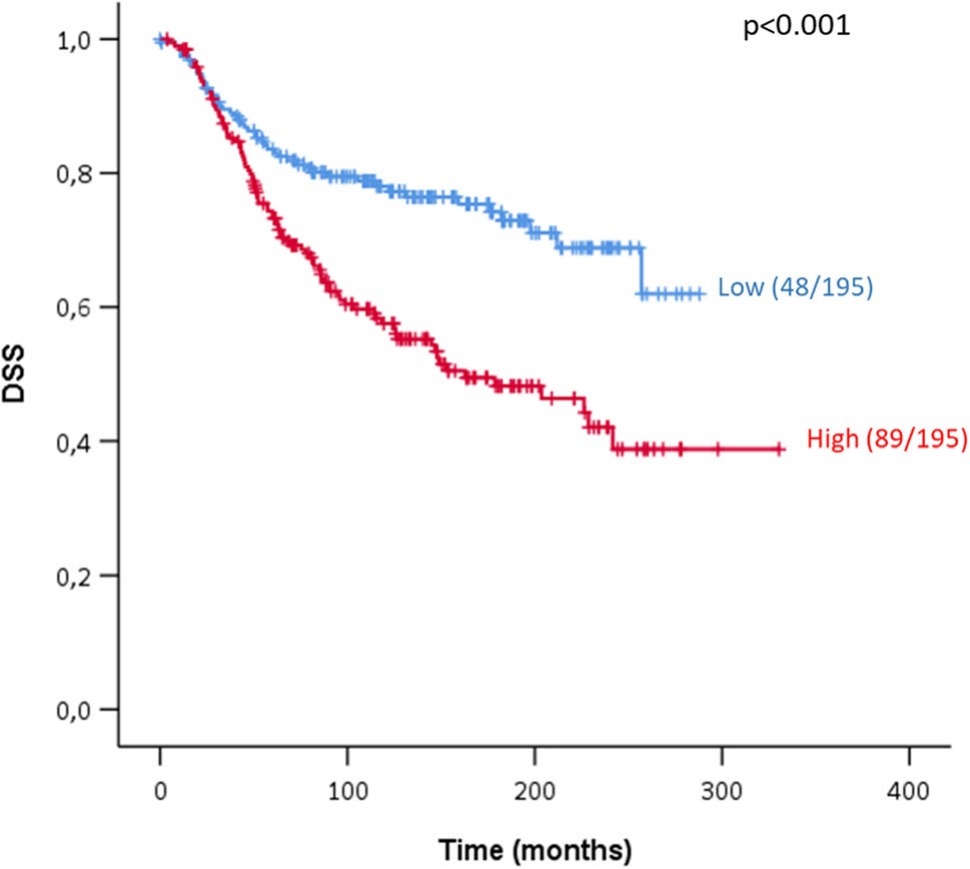
Patient survival depends on *ATOX1* mRNA expression level. Disease-specific survival (DSS) KM curves for breast cancer patients stratified by *ATOX1* expression. “Low” are the bottom 10% (*n* = 195) and “high” are the top 10% (*n* = 195) of the patient cohort (total *n* = 1904) after sorting patients based upon *ATOX1* mRNA expression levels

Upon survival analysis of all different breast cancer patients in the METABRIC cohort, we discovered that patients with high *ATOX1* mRNA levels (10% of patients with highest *ATOX1* mRNA levels, i.e., more than a 1.3-fold increase above median) in their primary tumor have poorer survival than those with low *ATOX1* mRNA levels (10% of patients with lowest *ATOX1* mRNA levels, i.e., more than a 1.3-fold decrease below median) (median DSS is 130 months for low *ATOX1* vs. 85 months for high *ATOX1*, *p* < 0.001, Fig. 1). Notably, the survival disadvantage is maintained when comparing the 10% of patients with highest *ATOX1* mRNA levels (*n* = 195) vs. all other 90% of patients in the cohort (*n* = 1709) (Fig. S1). Thus, *ATOX1* expression portends a survival disadvantage in breast cancer patients. Since survival is largely coupled to metastasis, high *ATOX1* levels may report on increased occurrence of metastasis in those patients.

Follow-up time for the selected cohort (*n* = 390, i.e., lowest 10% and highest 10% merged) was up to 330 months with a median of 119 months and number of disease-specific events (i.e., breast cancer death) was 137 out of 390 (35.1%) [death by other causes, 87 (22.3%)]. Other clinicopathological parameters that significantly variate between the low and high *ATOX1* expression groups are menopausal state (*p* = 0.024), histological grade (*p* = 0.044), lymph node status (*p* < 0.001), human epidermal growth factor receptor 2 (HER2) status (*p* < 0.001), estrogen receptor (ER) status (*p* = 0.023) and hormone therapy (*p* < 0.001) (Table S1). More, larger (> 2 cm) tumors (*p* = 0.017), histological grade 3 (*p* = 0.039), positive lymph nodes (*p* < 0.001), moderate and high cellularity (*p* < 0.001), and progesterone receptor (PR)-positivity (*p* = 0.001) correlate with worse disease-specific survival of patients in the selected cohort (Table S2). More, in Fig. S2 we present the number of patients with low (lowest 10%, *n* = 195) or high (highest 10%, *n* = 195) *ATOX1* mRNA expression as a function of PAM50 molecular subtype and tumor stage. We also included the numbers of patients who received hormone therapy and/or chemotherapy.

### High ATOX1 levels relate with worse survival of patients with specific breast cancer subtypes

To obtain more detailed information on the role of ATOX1 in breast cancer, we evaluated the correlation between *ATOX1* expression levels and survival outcome for the different PAM50 molecular subtypes, plus the Claudin low molecular subtype (see Fig.S3A, B for KM curves for DSS of patients for different subtypes of breast cancer within the entire cohort (*n* = 1904) and the selected cohort (*n* = 390), respectively). The PAM50 subtypes of breast cancer are based upon patterns of gene expression (i.e., 50-gene subtype predictor) and are defined into five groups of Luminal A, Luminal B, HER2-enriched, Basal-like, and Normal-like [12].

**Fig. 2:**
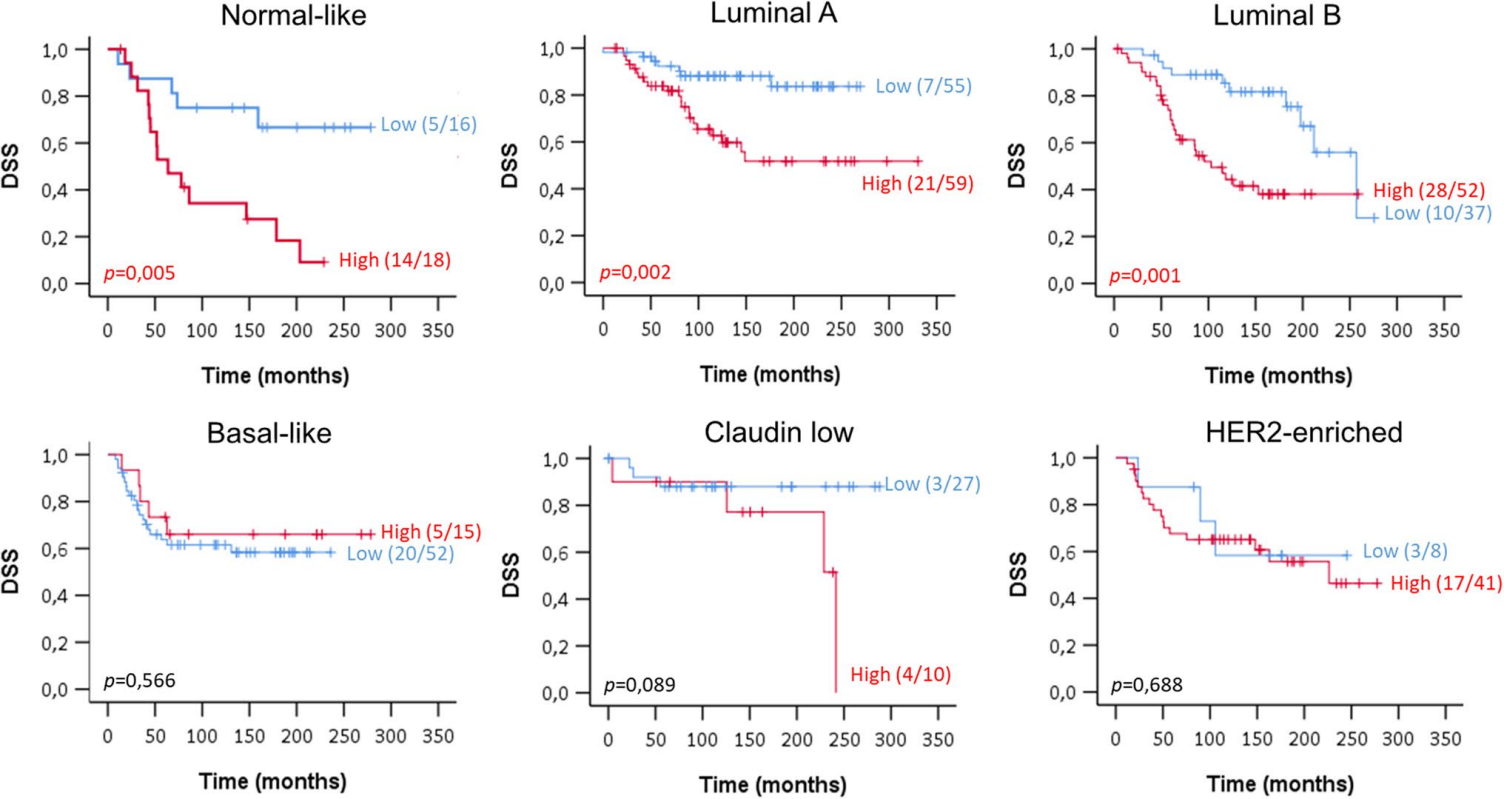
High *ATOX1* expression is correlated with worse survival outcome for specific subtypes of breast cancer disease. Disease-specific survival (DSS) KM curve for the different PAM50 molecular subtypes of breast cancer patients stratified by *ATOX1* expression. We observed a strong association between *ATOX1* levels and DSS for patients with Normal-like (*p* = 0.005), Luminal A (LumA) (*p* = 0.002) and Luminal B (LumB) (*p* = 0.001) breast tumors

We found a strong correlation between high *ATOX1* mRNA levels and reduced DSS for Normal-like (median DSS of 161 months for low *ATOX1* vs. 64 months for high *ATOX1*, *p* = 0.005), Luminal A (171 vs. 91 months, *p* = 0.002) and Luminal B (158 vs. 67 months, *p* = 0.001) breast cancer subtypes. We found no significant correlation between *ATOX1* mRNA levels and patient survival for Basal-like (79 vs. 76 months), Claudin low (110 vs. 105 months) and HER2-enriched cancer subtypes (106 vs. 82 months) [Fig. 2, Fig. S4 shows overall survival (OS) KM plots]. This demonstrates that the *ATOX1* expression level correlates with patient survival only in specific molecular subtypes of breast cancer.

### High ATOX1 mRNA levels relate with worse prognosis for patients at early stages of disease

**Fig. 3:**
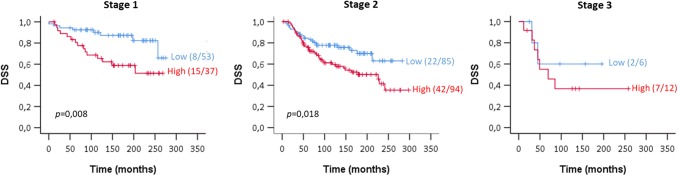
High *ATOX1* expression is correlated with worse patient survival at early stages of breast cancer disease. Disease-specific survival (DSS) KM curve for the different stages of breast cancer disease stratified by *ATOX1* expression. We observed a strong association for disease stage 1 (*p* = 0.008) and stage 2 (*p* = 0.018)

Next, we investigated *ATOX1* expression vs. survival outcome at different stages of breast cancer. While the primary tumor at stages 0 and 1 is localized, at stages 2 and 3 the tumor shows increased regional spread to the lymph nodes [13]. Interestingly, we found the largest correlation between *ATOX1* expression levels and disease-specific patient survival for stage 1 breast cancer tumors (median DSS of 164 months for low *ATOX1* vs. 100 months for high *ATOX1*, *p* = 0.008), followed by a less significant correlation for stage 2 tumors (131 vs. 90 months, *p* = 0.018) and no correlation for stage 3 tumors (Fig. 3). Since the number of stage 3 cases was very small (*n* = 18) in the selected cohort, we additionally evaluated the DSS considering the entire cohort (*n* = 1904) with comparison of 10% highest *ATOX1* expression group (*n* = 195) vs. the lower 90% of the patient cohort (*n* = 1709) (Fig. S5). Supportive, no significant correlation was detected between *ATOX1* expression and survival for stage 3 tumors (48 vs. 59 months). Additionally, considering the entire patient cohort, the difference in survival for stage 2 patients with low vs. high *ATOX1* levels became non-significant (*p* = 0.058). Stages 0 and 4 could not be assessed because there are only few patients with these stages of disease in the METABRIC data set. Thus, the *ATOX1* expression level appears to be a determinant of survival only at early stages of breast cancer.

## Discussion

We found that patients with high *ATOX1* expression levels in their primary tumor have approximately 50% lower survival chances (median DSS decreased by a factor of 2). This suggests that ATOX1 may participate in breast cancer-related processes leading to patient deaths. As 90% of all cancer-related deaths are due to metastasis [8], this finding suggests that ATOX1 may play a crucial role in processes facilitating breast cancer metastasis like cancer cell migration.

Evaluation of the different PAM50 molecular subtypes using the METABRIC breast cancer data set shows significant correlations between high *ATOX1* expression and decreased DSS for Luminal A, Luminal B and Normal-like breast tumors, but not for Claudin low, Basal-like and HER2-enriched tumors. However, the KM plots for OS demonstrate a significant relation between high *ATOX1* levels and worse survival of patients with Claudin low subtype tumors (Fig. S4), thus there may be a correlation in this subtype too if the patient number analyzed is increased. As different subtypes may be dominated by different oncogenic pathways, it is possible that ATOX1 acts in several cancer-promoting molecular paths leading to patient death. Nonetheless, cell migration, allowing for metastasis, is a common process in all subtypes and we earlier found that ATOX1 plays a role in breast cancer cell migration in cell culture studies [6], although the underlying pathways are not yet established.

Intriguingly, the data show *ATOX1* to have prognostic value in the hormone receptor-positive breast cancers only (and Claudin low, when considering OS). In accord, comparison of KM survival plots of the data divided into ER-positive and ER-negative tumors indicates significant worse survival for high *ATOX1* tumors that are ER-positive only (*p* < 0.001) (Fig. S6). A potential explanation for this distinction is that the increased *ATOX1* expression in ER-positive tumors influences mitogen-activated protein kinase (MAPK) signaling, perhaps via the ATOX1–ATP7A–lysyl oxidase (LOX) axis [6], which in turn promotes tumor growth and metastasis. In support of such a link, *ATOX1* knockout in B-Raf proto-oncogene, serine/threonine kinase (BRAF) mutation-positive melanoma cells was found to reduce MAPK signaling [14]. In ER-negative tumors, other pathways (not dependent on *ATOX1* levels) may instead define cancer progression [15]. We note that ER-negative and lymph node-positive tumors, but not ER-positive and/or lymph node-negative tumors, were treated with chemotherapy (before surgery) (Fig. S2). Thus, the correlation of high *ATOX1* levels with worse patient survival for ER-positive tumors only may relate to differences in treatment. Note, the evaluation of prognostic biomarkers using databases such as METABRIC and TCGA has to be carefully interpreted because information of the other prognostic variables such as stage and grade may lack in relatively a large proportion of patients.

Another important finding is that elevated *ATOX1* expression selectively correlates with poor survival for breast cancer stages 1 and 2, but not for stage 3. Likely, because stage 3 tumors have already spread, ATOX1 may not be of importance. In addition, at stage 3, the expression level of *ATOX1* is higher on average than in stages 1 and 2 (*p* = 0.037) (Fig. S7). Thus, variations between high and low levels may not matter at stage 3 as they may always be above a threshold. Notably, for stage 4, which lacked sufficient data for making a survival plot, the *ATOX1* level appears even higher (Fig. S7). The role of ATOX1 in metastasis is further supported by the observation of increased *ATOX1* levels in lymph node-positive tumors and the fact that there is a correlation between presence of positive lymph nodes and worse patient prognosis (Tables S1, 2).

Taken together, our data indicate that the *ATOX1* expression level can be used as a biomarker in early stages of breast cancer, whereby high *ATOX1* levels correlate with worse survival prognosis in Luminal A, Luminal B and Normal-like (and Claudin low when considering OS data) tumors. In such cases, Cu chelation therapy may be helpful to patients to prolong their lives. Further molecular-mechanistic studies of the underlying pathways, which result in *ATOX1* expression levels being correlated with breast cancer patient survival, may allow for the discovery of new cancer drug targets.

## Electronic supplementary material

Below is the link to the electronic supplementary material.

Supplementary file1 (DOCX 1311 kb)
