# Supplementary material for: Evaluation of copper chaperone ATOX1 as prognostic biomarker in breast cancer
Source: Breast Cancer. 2020 Jan 2;27(3):505–9. doi: 10.1007/s12282-019-01044-4 (PMC7196078; doi:10.1007/s12282-019-01044-4)
Supplement: Supplementary file 1 — Supplementary file1 (DOCX 1311 kb) [file 12282_2019_1044_MOESM1_ESM.docx]

**Supplementary material**

**Tables S1-2**

**Fig.S1-7**

**Table S1. Correlation between the clinicopathological characteristics and *ATOX1* expression levels.** All, the selected cohort of breast cancer patients (n=390); Low and High are bottom 10% (n=195) and top 10% (n=195) of the selected cohort after sorting patients based upon *ATOX1* mRNA expression levels, respectively.

| **Characteristics** | **All** | ***ATOX1*^Low^** | ***ATOX1*^High^** | ***p*-value** |
| --- | --- | --- | --- | --- |
| **Age at diagnosis (years)** |  |  |  |  |
| <61 | 193 (49,5%) | 100 | 93 |  |
| ≥61 | 197 (50,5%) | 95 | 102 |  |
| **Tumor Size (cm)** |  |  |  |  |
| ≤2 | 154 (39,5%) | 84 | 70 |  |
| 2,1-5 | 214 (54,9%) | 102 | 112 |  |
| >5 | 18 (4,6%) | 8 | 12 |  |
| unknown | 4 (1,0%) |  |  |  |
| **Tumor stage** |  |  |  |  |
| 0 | 0 (0%) |  |  |  |
| 1 | 90 (23,1%) | 53 | 37 |  |
| 2 | 179 (46,9%) | 85 | 94 |  |
| 3 | 18 (4,6%) | 6 | 12 |  |
| 4 | 1 (0,3%) | 0 | 1 |  |
| Unknown | 102 (26,2%) |  |  |  |
| **PAM50 molecular subtypes** |  |  |  |  |
| Normal-like | 34 (8,7%) | 16 | 18 | <0,001 |
| Luminal A | 114 (29,2%) | 55 | 59 |  |
| Luminal B | 89 (22,8%) | 37 | 52 |  |
| Basal-like | 67 (17,2%) | 52 | 15 |  |
| Claudin low | 37 (9,5%) | 27 | 10 |  |
| HER2-enriched | 49 (12,6%) | 8 | 41 |  |
| Unknown | 0 |  |  |  |
| **Type of Breast Surgery** |  |  |  |  |
| Mastectomy | 158 (40,5%) | 97 | 61 |  |
| Breast conserving | 232 (59,5%) | 98 | 134 |  |
| Unknown | 4 (0,5%) | 2 | 2 |  |
| **Inferred Menopausal State** |  |  |  |  |
| Pre- | 81 (20,8%) | 49 | 31 | 0,024 |
| Post- | 311 (79,7%) | 146 | 164 |  |
| **Cancer Type Detailed** |  |  |  |  |
| Breast Invasive Ductal Carcinoma | 322 (82,6%) | 155 | 167 |  |
| Breast Invasive Luminal Carcinoma | 24 (6,2%) | 16 | 8 |  |
| Mixed IDC and ILC | 32 (8,2%) | 14 | 18 |  |
| Mixed invasive mucinous carcinoma | 5 (1,3%) | 5 | 0 |  |
| Breast | 5 (1,3%) | 4 | 0 |  |
| Unknown | 3 (0,8%) | 1 | 2 |  |
| **Neoplasm Histologic Grade** |  |  |  |  |
| 1 | 23 (5,9%) | 16 | 7 | 0,044 |
| 2 | 133 (34,1%) | 71 | 62 |  |
| 3 | 222 (56,9%) | 100 | 122 |  |
| Unknown | 12 (3,1%) |  |  |  |
| **Lymph node status** |  |  |  |  |
| 0 | 211 (54,1%) | 121 | 90 | <0,001 |
| 1 to 3 | 123 (31,5%) | 59 | 64 |  |
| ≥ 4 | 56 (14,4%) | 15 | 41 |  |
| unknown | 0 |  |  |  |
| **Cellularity** |  |  |  |  |
| Low | 32 (8,2%) | 20 | 12 |  |
| Moderate | 133 (34,1%) | 59 | 74 |  |
| High | 214 (54,9%) | 107 | 107 |  |
| Unknown | 11 (2,8%) | 9 | 9 |  |
| **PR Status** |  |  |  |  |
| PR-positive | 165 (42,3%) | 91 | 74 |  |
| PR-negative | 225 (57,7%) | 104 | 121 |  |
| **HER2 Status** |  |  |  |  |
| HER2-positive | 50 (12,8%) | 10 | 40 | <0,001 |
| HER2-negative | 340 (87,2%) | 185 | 155 |  |
| **ER Status** |  |  |  |  |
| ER-positive | 263 (67,4%) | 121 | 142 | 0,023 |
| ER-negative | 127 (32,6%) | 74 | 121 |  |
| **Radiation therapy** |  |  |  |  |
| Yes | 239 (61,3%) | 117 | 122 |  |
| No | 151 (38,7%) | 78 | 73 |  |
| **Chemotherapy** |  |  |  |  |
| Yes | 107 (27,4%) | 54 | 53 |  |
| No | 283 (72,6%) | 141 | 142 |  |
| **Hormone Therapy** |  |  |  |  |
| Yes | 235 (60,3%) | 100 | 135 | <0,001 |
| No | 155 (39,7%) | 95 | 60 |  |
| **Vital status of patient (n)** |  |  |  |  |
| Alive | 166 (42,6%) | 111 | 55 |  |
| Death by breast cancer | 137 (35,1%) | 48 | 89 |  |
| Death by other causes | 87 (22,3%) | 36 | 51 |  |
|  |  |  |  |  |
|  |  |  |  |  |
|  |  |  |  |  |
|  |  |  |  |  |
|  |  |  |  |  |
|  |  |  |  |  |

| Table S2. Other clinicopathological parameters influencing prognosis of breast cancer patients (n=390). | | |
| --- | --- | --- |
| **Clinicopathological parameters** | ***p*-value** | **HR (95 % CI)** |
| **Age** (≥ 61 *vs.* <61 years) | 0,995 | 0,999(0,714-1,398) |
| **Tumor size** (>2 *vs.* ≤2 cm) | **0,017** | 1,547(1,082-2,213) |
| **Menopausal state** (post- *vs.* pre-) | 0,298 | 0,812(0,548-1,202) |
| **Histological grade** (III *vs.* I/II) | **0,039** | 4,351(1,076-17,583) |
| **Lymph node status** (yes *vs.* no) | **<0,001** | 1,866(1,504-2,314) |
| **Cellularity** (low *vs.* moderate/high) | **<0,001** | 1,982(1,393-2,820) |
| **ER status** (+ *vs.* -) | 0,710 | 0,727(0,514-1,028) |
| **PR status** (+ *vs.* -) | **0,001** | 0,548(0,383-0,785) |
| **HER2 status** (+ *vs.* -) | 0,292 | 1,291(0,803-2,076) |

*HR: hazard ratio; CI: confidence interval*


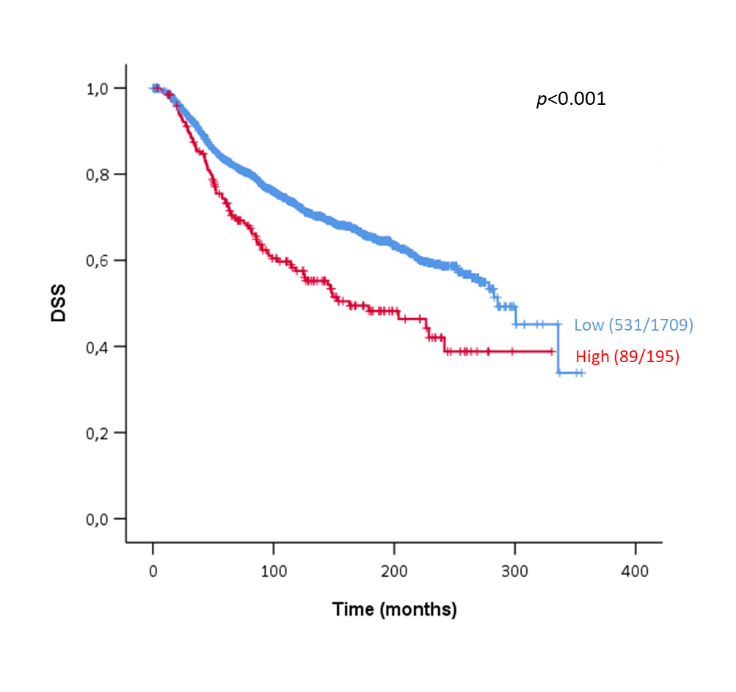


Fig.S1. Kaplan-Meier curves with disease-specific survival (DSS) of breast cancer patients stratified by *ATOX1* mRNA expression. “Low” are the bottom 90% (n=1709) and “high” are the top 10% (n=195) of the entire patient cohort (n=1904) after sorting patients based upon *ATOX1* mRNA expression levels. P-value is presented for the log-rank statistical test.


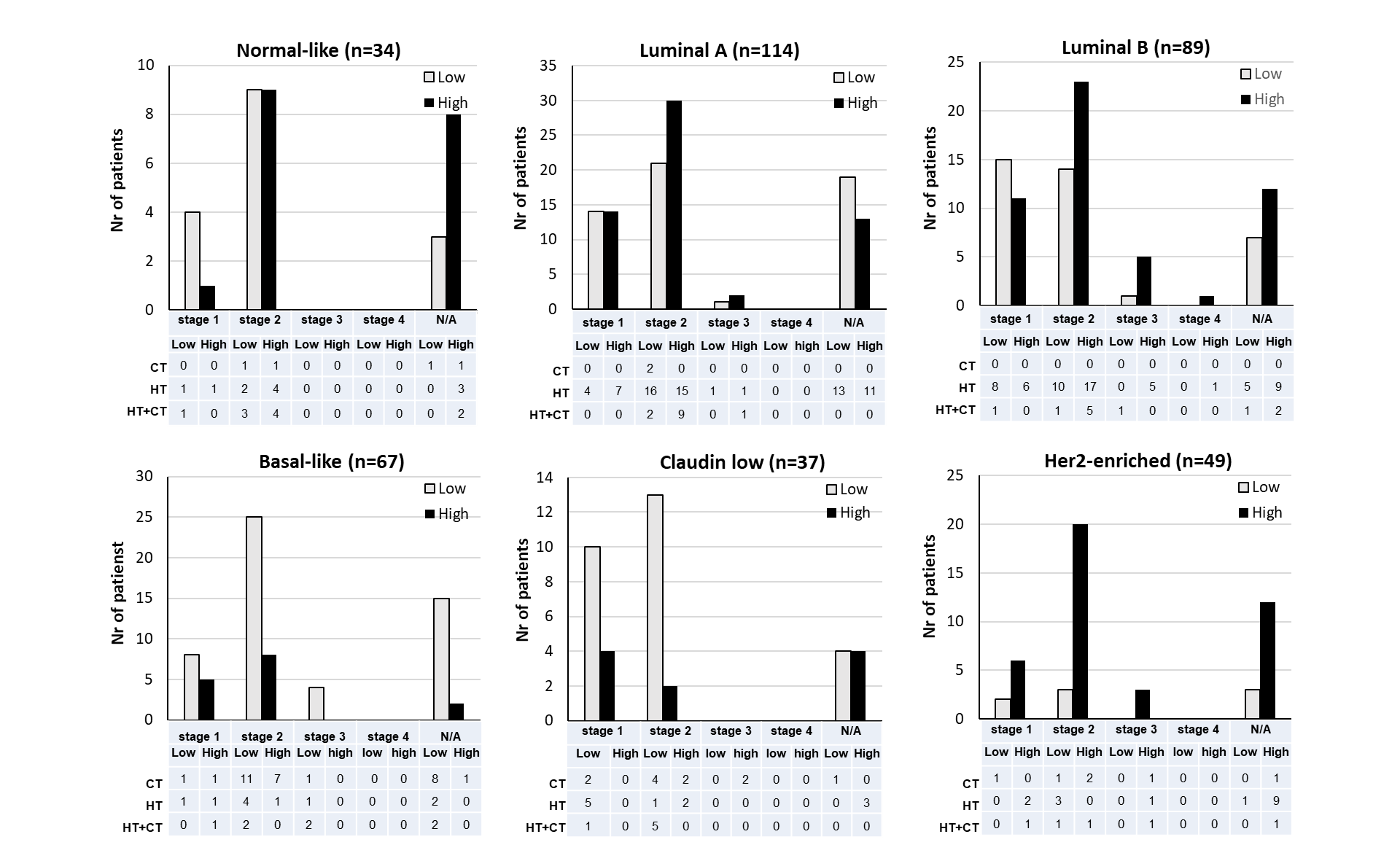


Fig.S2. Analysis of the number of patients with low (10%, n=195) and high (10%, n=195) *ATOX1* mRNA expression within the six different PAM50 molecular subtypes by tumor stage and included whether the patients were treated with hormone therapy (HT) and/or chemotherapy (CT).


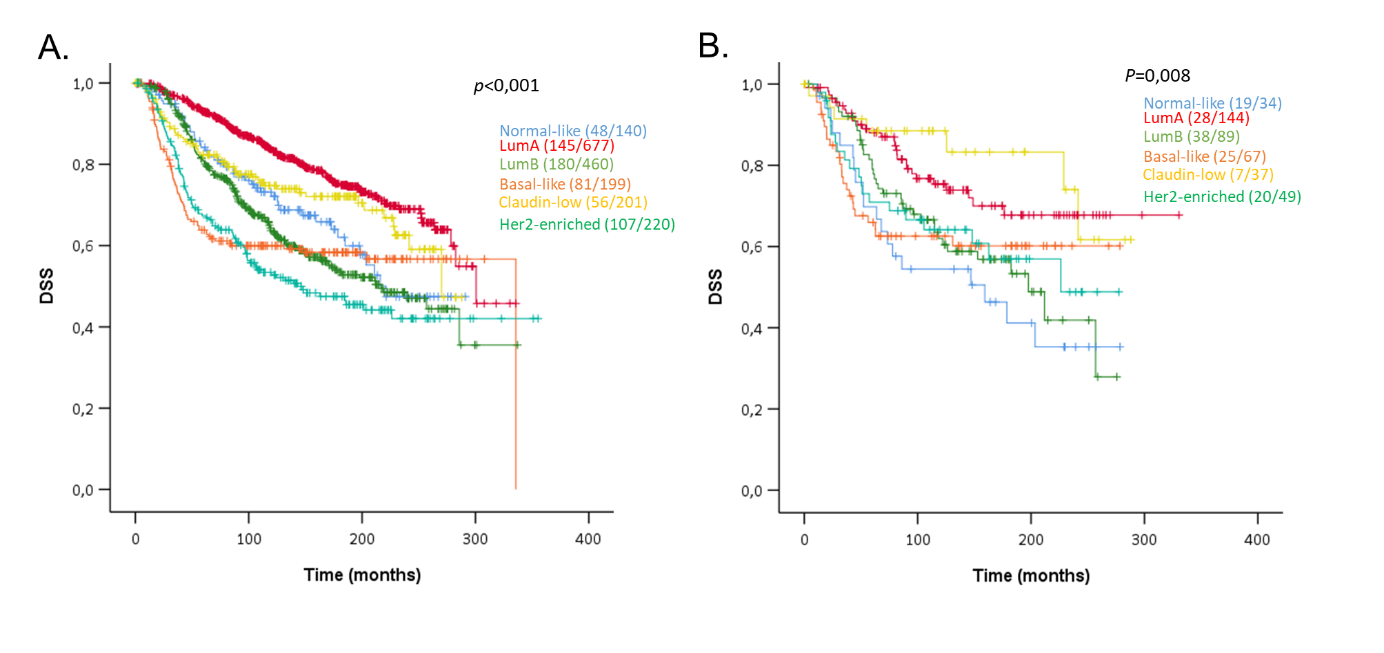


Fig.S3. Kaplan-Meier curves with disease-specific survival (DSS) of the patients stratified by the six different PAM50 molecular subtypes (Normal-like, Luminal A (LumA), Luminal B (LumB), Basal-like, Claudin low and HER2-enriched) in (A) the entire cohort (n=1904) and (B) the selected cohort (n=390). P-value is given for the log-rank statistical test.


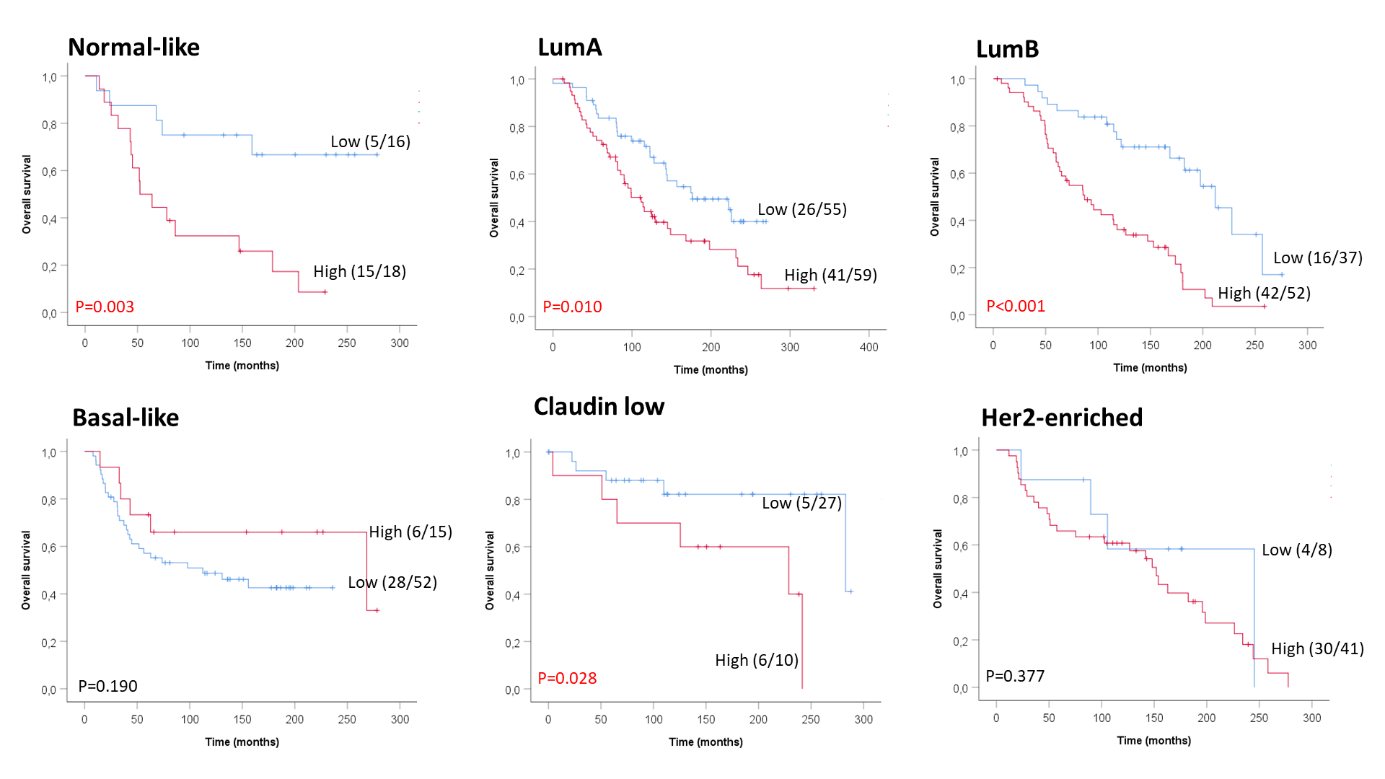


Fig.S4. Kaplan-Meier plots with overall survival (OS) plots for breast cancer patients with low versus high *ATOX1* expression levels in their primary tumor stratified for the different PAM50 molecular subtypes stratified by *Atox1* expression levels. “Low” are the bottom 10% (n=195) and “high” are the top 10% (n=195) of the selected patient cohort (n=390) after sorting patients based upon *ATOX1* mRNA expression levels. Note, for the Claudin low subtype we observe a significant correlation between high *ATOX1* levels and worse OS. P-value is given for the log-rank statistical test.


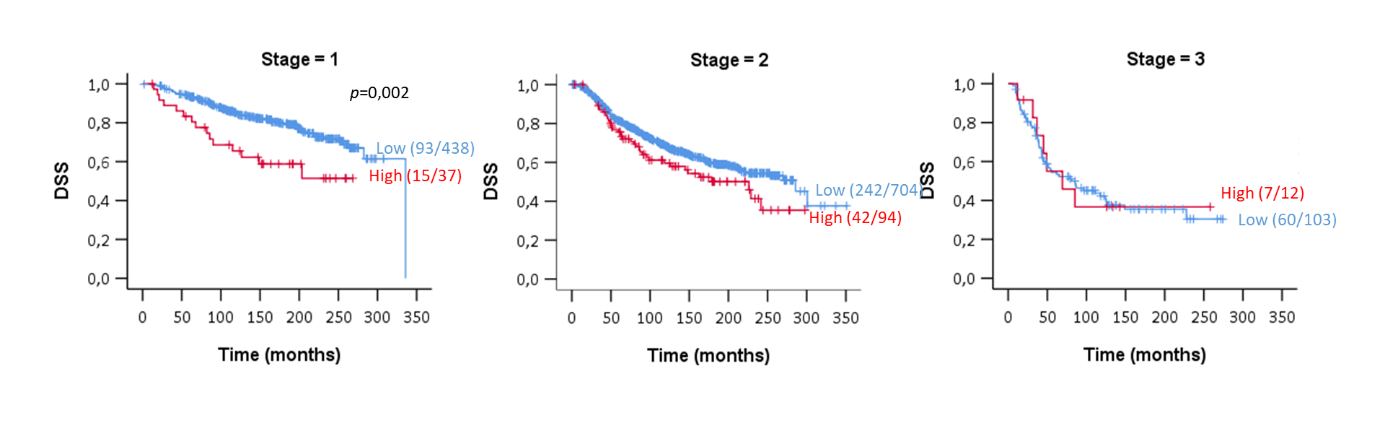


Fig.S5. Kaplan-Meier plots with disease-specific survival (DSS) of breast cancer patients (n=1904) at different stages of disease with comparison of low *ATOX1* (90%, n=1709) and high *ATOX1* (10%, n=195). P-value is presented for the log-rank statistical test.


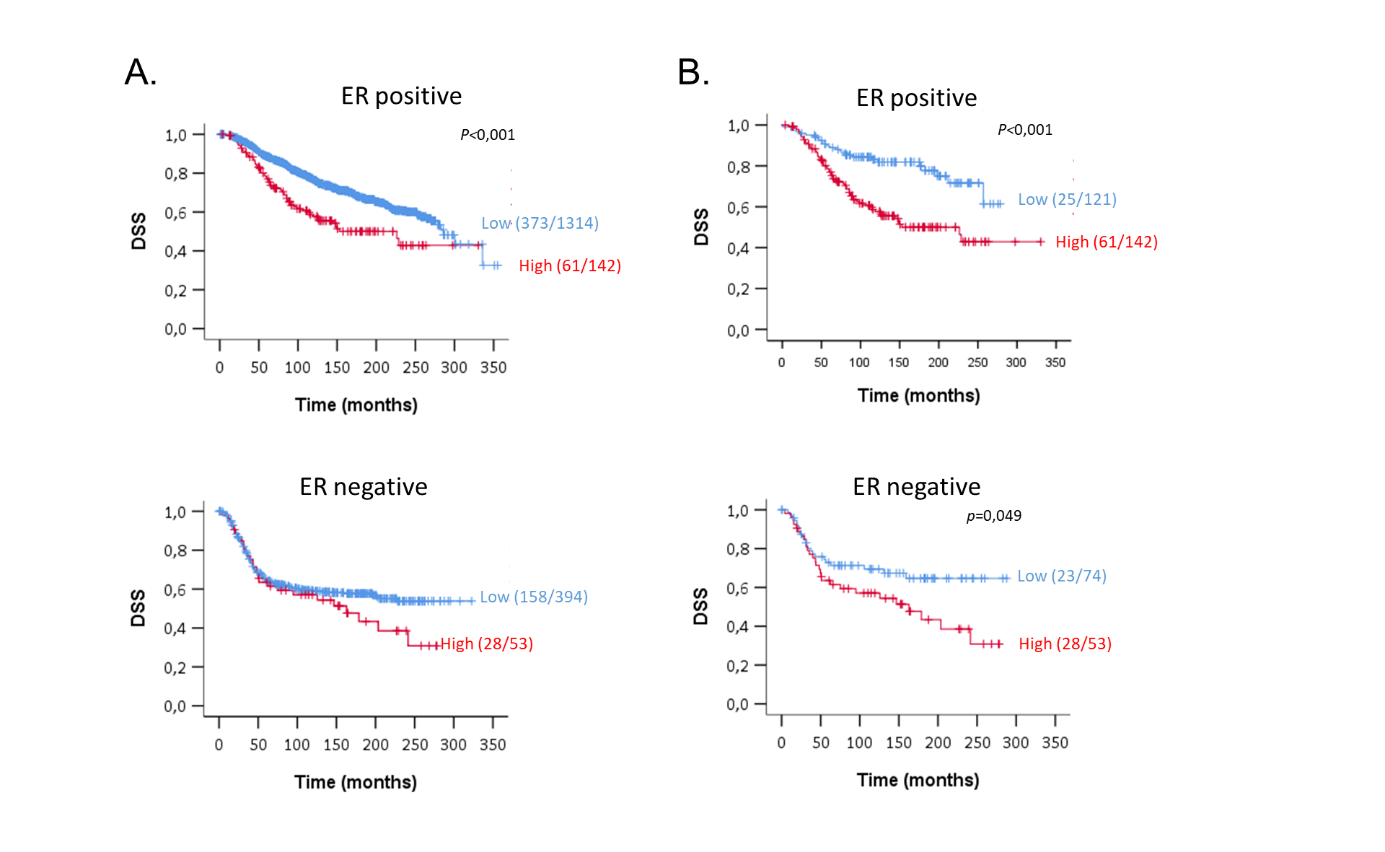


Fig.S6. Kaplan-Meier plots with disease-specific survival (DSS) of patients with ER-positive versus ER-negative breast tumors with comparison of low *ATOX1* versus high *ATOX1* expression levels using (A) the entire cohort (n=1904) or (B) the selected cohort (n=390).


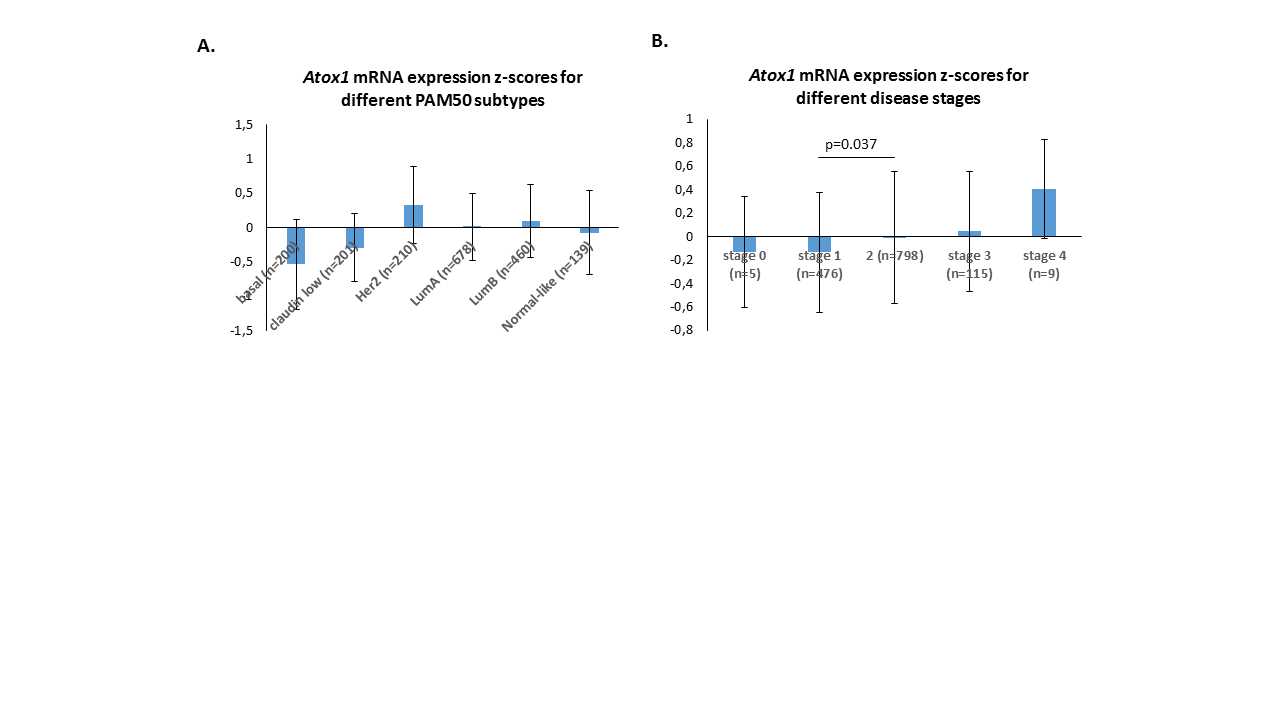


Fig.S7. *ATOX1* expression levels in primary tumors of breast cancer patients with (A) different PAM50 molecular subtypes and (B) different stages of disease in the entire cohort (n=1904). Error bars indicate the standard deviation of the mean. P-value is for student´s t-test.
